# Molecular Modulation of Threadfin Fish Brain to Hypoxia Challenge and Recovery Revealed by Multi-Omics Profiling

**DOI:** 10.3390/ijms26041703

**Published:** 2025-02-17

**Authors:** Xiaoli Ma, Wen-Xiong Wang

**Affiliations:** 1 School of Energy and Environment and State Key Laboratory of Marine Pollution, City University of Hong Kong, Kowloon, Hong Kong, China; xiaolima@cityu.edu.hk; 2 Research Centre for the Oceans and Human Health, City University of Hong Kong Shenzhen Research Institute, Shenzhen 518057, China

**Keywords:** *Eleutheronema tetradactylum*, hypoxia, recovery, brain damage, multi-omics sequencing

## Abstract

Migratory fish often encounter hypoxic zones during migration, which can lead to varying degrees of hypoxic stress. This issue has become increasingly severe due to human activities and climate change, which have resulted in the expansion of hypoxic zones in aquatic environments. However, there is limited research on how these species respond to hypoxic stress and subsequent recovery. In this study, we used *Eleutheronema tetradactylum*, a well-recognized migratory and economically valuable fish species, as a model organism. Histological analysis revealed extensive neuronal damage during hypoxia exposure, with limited recovery observed even after 12 h of reoxygenation. Differential gene expression analysis highlighted progressive alterations in genes associated with stress response, neuroactive ligand interactions, and cellular repair mechanisms. Time-series analysis of differentially expressed genes (DEGs) identified critical expression profiles throughout the hypoxia-recovery process and revealed hub genes for each stage. Furthermore, dynamic changes in miRNA expression and proteomic profiles indicated active regulation of several key biological pathways, including MAPK, HIF-1, and ECM-receptor interactions. Through miRNA-mRNA-protein correlation analysis, we propose a model that predicts key regulatory pathways and critical miRNA-mRNA-protein interactions across the various stages of hypoxia-recovery in the brain of *E. tetradactylum*. This study presents the first integrated analysis of miRNA, mRNA, and protein throughout the entire hypoxia-recovery process in fish brains. The molecular interactions and regulatory pathways identified in this model could serve as valuable biomarkers for future research on hypoxia-recovery mechanisms in fish.

## 1. Introduction

Dissolved oxygen (DO) is a fundamental environmental factor for fish survival. Hypoxic conditions can disrupt fish physiological and biochemical enzyme activities, gene expression, as well as signaling pathways, thereby affecting their behavioral activities, growth, development, and metabolic processes and ultimately altering the energy flow and ecological characteristics of the entire population [1,2]. Global warming leads to rising sea temperatures, which decreases the capacity of water to dissolve oxygen [3]. Additionally, the discharge of fertilizers, sewage, and animal waste into the oceans provides abundant nutrients that promote the excessive growth of algae [4]. When combined with air pollution, this results in a significant depletion of dissolved oxygen in marine environments, creating hypoxic zones, often referred to as “dead zones”, where the available habitat for marine life is further constrained. In water layers between 300 and 700 m deep in the equatorial Pacific and tropical Atlantic oceans, oxygen concentrations decrease by 0.09 to 0.34 micromoles per kilogram annually [5]. Coastal areas in Asia, including Hong Kong, are also among the regions most severely affected by this trend [3]. In freshwater ecosystems, human activities also contribute to nutrient enrichment and organic matter influx into estuaries, leading to eutrophication. The subsequent decomposition of organic material further depletes dissolved oxygen, exacerbating hypoxic conditions in certain freshwater areas [3,6]. For migratory fish, encounters with hypoxic zones during migration could lead to varying degrees of hypoxic stress [7]. Understanding the capacity of these species to recover from hypoxic damage upon reoxygenation is crucial, as it provides valuable insights into both the short-term and long-term effects of hypoxia on fish populations.

The brain is particularly sensitive to fluctuations in environmental oxygen levels compared to other organs. Although it comprises only 2–3% of total body weight, the brain consumes 20–30% of the body’s oxygen [8]. In contrast to mammals, fish are more frequently and intensely exposed to variations in dissolved oxygen levels in aquatic environments, making their brains more susceptible to hypoxic damage [9]. Hypoxia can lead to irreversible pathological alterations in brain cells, resulting in acidosis, oxidative damage, impaired energy supply, and decreased regulatory function of the cerebral vasculature [10]. Furthermore, studies have shown that hypoxia can impair the formation of intercellular networks within the central nervous system and reduce neuronal signal transmission, thus lowering action potentials and synaptic plasticity [11]. Oxygen supplied to the brain is primarily utilized for ATP production, which is essentially for maintaining synaptic and action potential signaling [12,13]. Additionally, the brain has minimal energy reserves and relies entirely on blood circulation to supply the necessary nutrients and oxygen to support survival and normal physiological functions [14]. Research has indicated that short-term hypoxia stress can cause significant neurological damage, while prolonged or severe hypoxia could alter reactive oxygen species (ROS) levels, leading to oxidative stress, loss of cellular function, and eventually cell apoptosis or necrosis [15,16]. Prolonged hypoxia has been shown to significantly reduce lipid peroxidation and antioxidant enzyme activity in the brains of *Perccottus glenii* [17]. Moreover, Johansson and Nilsson’s studies demonstrated that under extreme hypoxia conditions, the brains of *Carassius carassius* L. relied entirely on glycolysis, with a dramatic decrease in ATP levels and energy charge [18]. However, these studies primarily focused on initial responses and employed single-method sequencing techniques. The biological responses triggered by hypoxia involve a complex network of regulatory mechanisms that require further in-depth investigation.

Multi-omics analysis offers a significant advantage by integrating information across various omics levels, enabling a comprehensive exploration of key regulatory factors. For instance, an integrated analysis comprising the transcriptome, proteome, and metabolome in yellow catfish *(Pelteobagrus fulvidraco*) revealed common cerebral responses to chronic hypoxia, such as the activation of HIF signaling pathways, angiogenesis, and enhanced erythrocyte oxygen transport, with males and females exhibiting distinct cerebral response mechanisms to hypoxia [19]. In research on darkbarbel catfish (*Pelteobagrus vachelli*) under hypoxic stress, comprehensive sequencing of mRNA, miRNA, protein, and metabolites identified several hypoxia-related biomarkers that could serve as potential indicators for hypoxia studies in fish [20]. However, those studies only focused on the brain responses under hypoxia stress using the multi-omics method but ignored their recovery ability and mechanisms. To the best of our knowledge, multi-omics studies on the brain during the entire hypoxia-reoxygenation process in fish remain scarce.

The threadfin fish (*Eleutheronema tetradactylum*) is a typical migratory species that inhabits sandy coastal areas and occasionally migrates into estuaries or mangrove forests for feeding [21]. They undertake migrations to harbors during the breeding season, returning to the open sea post-spawning [22]. *E. tetradactylum* is also a commercially significant species in global aquaculture, known for its flavorful flesh, rich in unsaturated fatty acids and other nutrients [23]. This species is highly sensitive to hypoxic environments and can suffer rapid mortality when exposed to water [24]. Its pronounced intolerance to hypoxia, coupled with its migratory behavior, makes it an ideal model for investigating the intrinsic mechanisms of fish during the hypoxia-recovery process. In this study, we employed a multi-omics approach, integrating transcriptome (mRNA and miRNA) and proteome analyses of *E. tetradactylum* brains at different stages of the hypoxia-recovery process, providing a comprehensive examination of the biological responses and underlying mechanisms during the hypoxia-reoxygenation phase.

## 2. Results and Discussion

### 2.1. Morphological Alterations in the Threadfin Fish Brain During Hypoxia and Recovery

**Figure 1:**
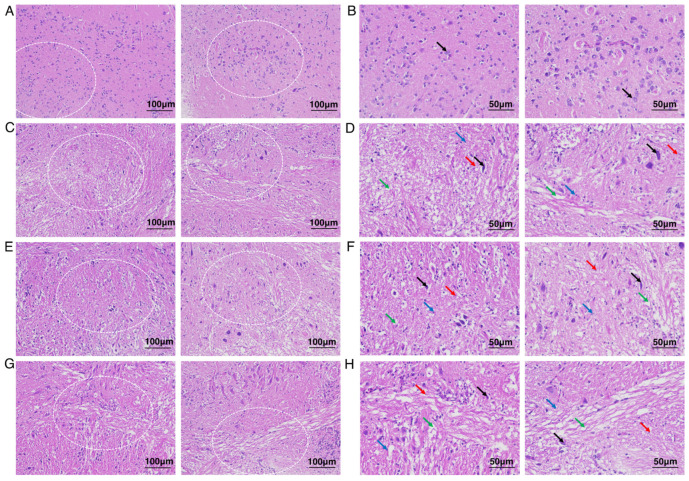
Histological analysis of *Eleutheronema tetradactylum* brain under hypoxia and recovery phases. (**A**,**B**) Histological characteristics of the brain in the control group. (**C**,**D**) Histological characteristics of the brain in the hypoxia group. (**E**,**F**) Histological characteristics of the brain in 6-h recovery group. (**G**,**H**) Histological characteristics of the brain in a 12-h recovery group. (**A**,**C**,**E**,**G**) ×200 magnification, scale bar = 100 μm; (**B**,**D**,**F**,**H**) ×400 magnification, scale bar = 50 μm. (**B**,**D**,**F**,**H**) represent enlarged views of (**A**,**C**,**E**,**G**), respectively. The enlarged areas in (**A**,**C**,**E**,**G**) are indicated by white dashed lines. Black and red arrows indicate neurons, blue arrow represent glial cells, green arrow illustrates nerve fibers.

Hematoxylin and eosin (H&E) staining was employed to assess the histological changes in the brain of *E. tetradactylum* during the hypoxia-recovery process. As illustrated in Figure 1A,B, under normoxic conditions with a dissolved oxygen concentration of 6.5 mg/L, the brain exhibited a high density of neurons (indicated by black arrows), characterized by regular cell morphology and tightly and neatly arranged nerve fibers. Furthermore, no obvious proliferation of glial cells, necrosis, or inflammatory changes were detected. In contrast, after 12 h of hypoxia exposure, with a dissolved oxygen level of 2 mg/L (Figure 1C,D), the brain tissue displayed disordered, with extensive neuronal necrosis (red arrows), nuclear pyknosis and fragmentation, as well as some neuronal shrinkage (black arrows). The number of glial cells increased (blue arrows), and numerous nerve fibers showed swelling (green arrows) and vacuolization. In the 6-h (Figure 1E,F) and 12-h (Figure 1G,H) recovery groups, where the dissolved oxygen level was restored to 6.5 mg/L, the tissue exhibited alterations similar to those observed in the hypoxia group, with no significant recovery detected. This suggested that 12 h may be insufficient for the brain tissue of *E. tetradactylum* to fully recover to its baseline condition. Collectively, these morphological alterations observed during the hypoxia-recovery process in the brain of *E. tetradactylum* triggered our interest in further exploration of the underlying molecular mechanisms.

### 2.2. Comprehensive mRNA Expression Profiling Across the Hypoxia-Recovery Phages

In order to identify the changes in mRNA expression in the brain of *E. tetradactylum* during the hypoxia-reoxygenation process, we performed paired-end sequencing (PE150) using the Illumina NovaSeq 6000 platform in Biomarker Technologies Co., Ltd (Beijing, China). A total of 12 cDNA libraries were constructed, representing four experimental groups: the normal oxygen control group (NorR1, NorR2, NorR3), the hypoxia exposure group (HypoR1, HypoR2, HypoR3), the 6-h recovery group (Oxy6R1, Oxy6R2, Oxy6R3), and the 12-h recovery group (Oxy12R1, Oxy12R2, Oxy12R3). A statistical summary of the mRNA sequencing and assembly results is presented in Table S1, including total clean reads and the mapping rate to the reference genome. Expression variability across samples was visualized with box plots (Figure S1A), and principal component analysis (PCA) was performed to show the variation among the samples (Figure S1B).

**Figure 2:**
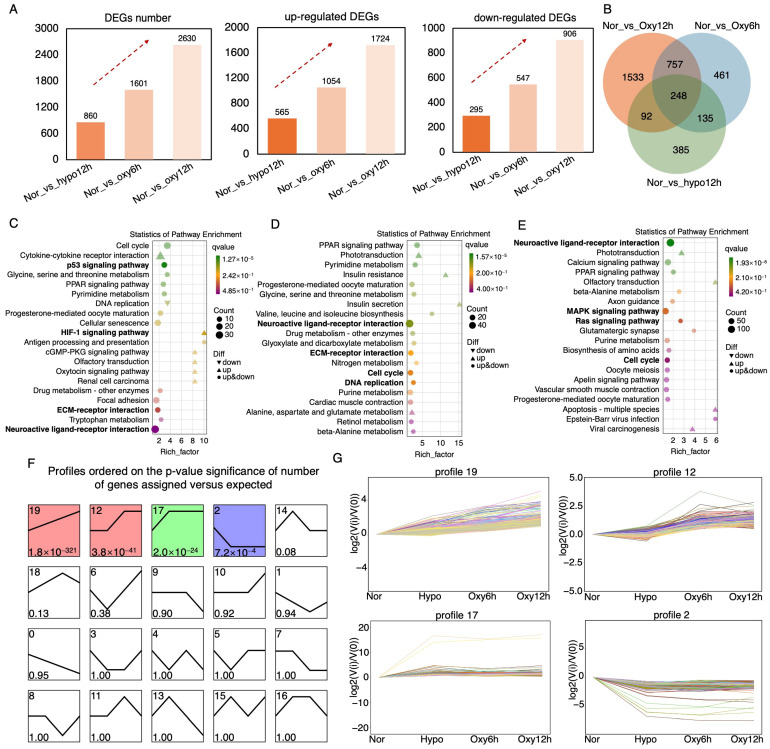
Comprehensive characterization of mRNA analysis during the hypoxia-recovery process in *Eleutheronema tetradactylum* brain. (**A**) Number and trend of differentially expressed genes (DEGs) across the hypoxia-recovery phages. (**B**) Integrated comparative analysis of the DEGs in the hypoxia-recovery process. KEGG pathway enrichment of the DEGs after (**C**) 6-h hypoxia, (**D**) 6-h recovery, and (**E**) 12-h recovery. (**F**,**G**) Clustering and trend analysis of DEGs across hypoxia-recovery phases, showing 20 enriched profiles. Profiles 19, 12, 17, and 2 exhibit significant enrichment (*p* < 0.05).

Differential expression analysis revealed that the number of differentially expressed genes (DEGs) in the hypoxia, 6-h recovery, and 12-h recovery groups relative to the control group were 860, 1601, and 2630, respectively, exhibiting an increasing trend. Of these, 565, 1054, and 1724 DEGs were upregulated, while 295, 547, and 906 were down-regulated (Figure 2A). These findings indicate a progressive accumulation of DEGs throughout the hypoxia-reoxygenation process, suggesting that after 12 h of reoxygenation, the brain of *E. tetradactylum* had not fully restored its baseline mRNA expression levels. A Venn diagram was generated to visualize the overlap of DEGs across different comparison groups (Figure 2B). The analysis revealed that 248 DEGs were consistently and differentially expressed across all hypoxia-reoxygenation stages when compared to the control group. Additionally, 385, 461, and 1533 DEGs were uniquely expressed in the hypoxia, 6-h recovery, and 12-h recovery stages, respectively. These results further suggested that distinct genes were involved at different stages of the hypoxia-reoxygenation process in the *E. tetradactylum* brain.

KEGG enrichment analysis of DEGs across different stages of the hypoxia-reoxygenation process revealed significant enrichment of the HIF-1 signaling pathway during the hypoxia stage. This pathway is well-known for regulating cellular responses to hypoxia by activating the expression of genes involved in angiogenesis, erythropoiesis, metabolism, and cell survival under low oxygen conditions [25]. Additionally, the p53 signaling pathway, which is crucial for regulating the cell cycle, apoptosis, and DNA repair in response to cellular stress, including DNA damage, was also significantly enriched (Figure 2C) [26,27]. The Neuroactive ligand-receptor interaction pathway was significantly enriched at all stages of hypoxia-reoxygenation in the *E. tetradactylum* brain. This pathway facilitates communication between neuroactive ligands (such as neurotransmitters, hormones, and peptides) and their respective receptors, playing an essential role in neural communication, neurotransmission, synaptic plasticity, and other neurophysiological processes [28,29]. Moreover, significant enrichment of the cell cycle pathway was observed at both the 6-h and 12-h reoxygenation stages, indicating active cellular repair during these phases (Figure 2D) [30]. At the 12-h reoxygenation stage, both the mitogen-activated protein kinase (MAPK) pathway, which regulates growth, differentiation, inflammation, and stress responses, and the Ras signaling pathway, which influences cell growth, differentiation, and survival, were significantly enriched (Figure 2E) [31,32].

To further elucidate the expression patterns of genes at different stages of hypoxia-reoxygenation and to identify key genes at each stage, we performed a time-series analysis of the DEGs. The DEGs were clustered based on their expression patterns throughout the hypoxia-reoxygenation process, revealing significant enrichment in profiles 19, 12, 17, and 2 (Figure 2F). The number of DEGs in each profile is shown in Figure S2, with the composition and functional annotation of these profiles detailed in Table S2. Profile 19, comprising 539 DEGs, represents the largest group of genes with consistently upregulated expression throughout the hypoxia-recovery process, suggesting their involvement across the entire hypoxia-reoxygenation cycle. Notable DEGs with the greatest fold changes in this profile include guanylate cyclase activator 1a (*guca1a*) and potassium voltage-gated channel subfamily V member 2-like (*LOC111585952*), which are predicted to play roles in retinal photoreceptor recovery and potassium ion transmembrane transport, respectively (Table S2) [33,34]. In profile 12, DEGs exhibited no change during the hypoxia phase but increased during the 6-h recovery stage, indicating their closer association with the recovery process. The DEGs with the largest fold changes in this profile include retinal cone rhodopsin-sensitive cGMP 3′,5′-cyclic phosphodiesterase subunit gamma (*LOC110002783*), a gene reported to be closely linked to retinal function (Table S2) [35]. This suggests that the brain of *E. tetradactylum* may activate genes related to retinal photoreception during hypoxia-reoxygenation, although further research is needed to confirm this hypothesis. In profiles 17 and 2, the DEGs displayed increasing and decreasing expression trends during the hypoxia stage, respectively, with no significant changes during the recovery phases. These results imply that these DEGs are more strongly associated with hypoxic stress, with profile 17 showing upregulation of DEGs during hypoxia and profile 2 showing down-regulation. Notably, the DEGs with the greatest fold changes in profile 2 include hemoglobin embryonic subunit alpha-like (*LOC115047007*) and hemoglobin subunit beta-2-like (*hbbe1.1*), which have been implicated in responses to hypoxic stress (Table S2) [36,37]. The reasons for the suppressed expression of these genes in the brain during hypoxia in *E. tetradactylum* remain unclear and warrant further investigation.

### 2.3. Dynamic microRNA Modulation Throughout Hypoxia-Recovery Process

**Figure 3:**
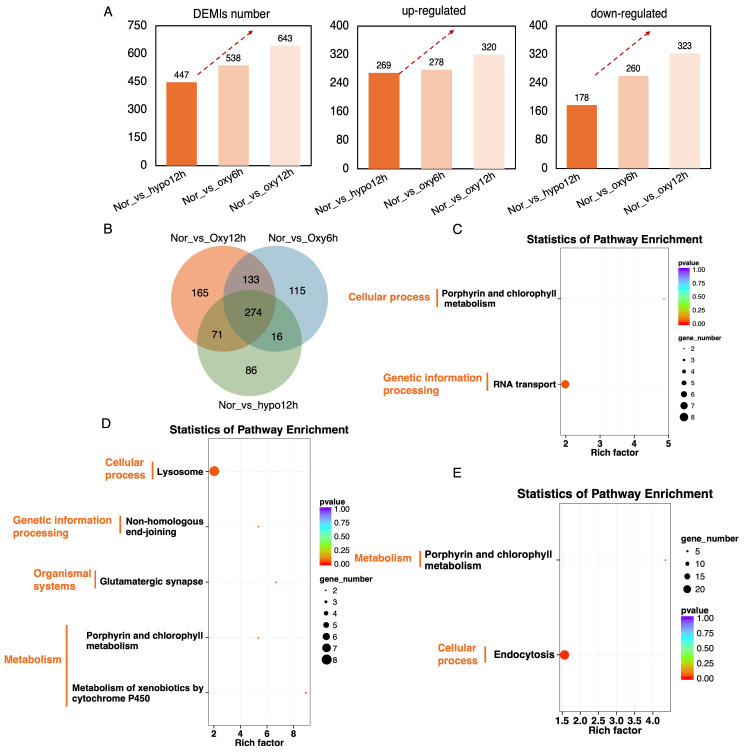
Overview of miRNA dynamics during the hypoxia-recovery process in *Eleutheronema tetradactylum* brain. (**A**) Number and trend of differentially expressed miRNAs (DEMIs) across the hypoxia-recovery phages. (**B**) Integrated comparative analysis of the DEMIs among hypoxia-recovery process. KEGG pathway enrichment of the DEMIs after (**C**) 6-h hypoxia, (**D**) 6-h recovery, and (**E**) 12-h recovery.

MicroRNA sequencing was conducted on 12 samples representing different stages of hypoxia-recovery in the brain of *E. tetradactylum*, identifying a total of 3965 miRNAs, which included 2983 known miRNAs and 982 novel miRNAs. The length distributions of both known and novel miRNAs were presented in Figure S3A,B. The overall expression distribution of miRNAs across samples is illustrated in the box plots in Figure S3C, while the clustering diagram comparing miRNA profiles among the different samples is shown in Figure S3D. A total of 6636 miRNA target genes were predicted, and a statistical summary of the miRNA data is provided in Table S3. Differential expression analysis of microRNAs (DEMIs) was conducted across the hypoxia, 6-h reoxygenation, and 12-h reoxygenation, compared to the control groups, with a total of 447, 538, and 643 DEMIs identified, respectively. Among these, 269, 278, and 320 DEMIs were upregulated, while 178, 260, and 323 DEMIs were down-regulated (Figure 3A), revealing an overall increasing trend during the hypoxia-recovery process. To further illustrate the overlap of DEMIs among the different comparison groups, a Venn diagram was generated, with 86, 115, and 165 DEMIs uniquely expressed in the hypoxia, 6-h recovery, and 12-h recovery stages, respectively (Figure 3B), suggesting that distinct miRNAs regulate different phases of the hypoxia-reoxygenation process in the brain of *E. tetradactylum*. Additionally, functional enrichment analysis of the target genes associated with differentially expressed miRNAs was performed. As shown in Figure 3C–E, significantly enriched pathways during the hypoxia-reoxygenation process were identified, with many of their top categories associated with cellular processes, genetic information processing, and metabolism, among others.

### 2.4. Proteomic Insights into the Hypoxia-Recovery Responses in Threadfin Fish

**Figure 4:**
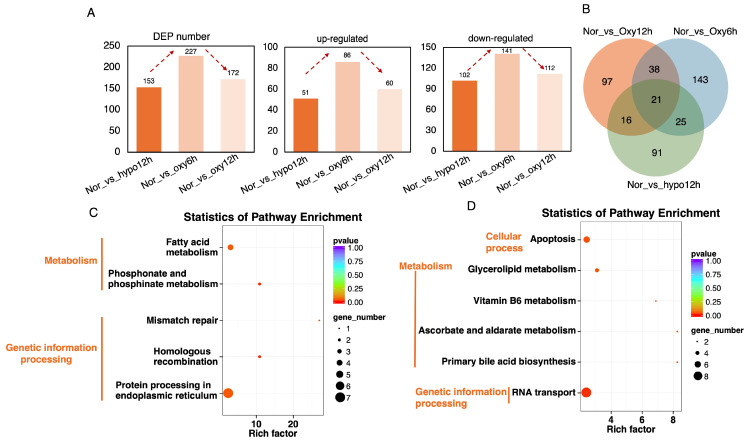
Characterization of protein dynamics during the hypoxia-recovery process in *Eleutheronema tetradactylum* brain. (**A**) Number and trend of differentially expressed proteins (DEPs) across the hypoxia-recovery phages. (**B**) Integrated comparative analysis of the DEPs among hypoxia-recovery process. KEGG pathway enrichment of the DEPs after (**C**) 6-h hypoxia, (**D**) 6-h recovery.

Proteomic analysis was conducted on 12 samples during the hypoxia-recovery processes, and the proteins were annotated using the GO, KEGG, and COG databases; a total of 4171 proteins were identified (Table S4). Based on the GO, KEGG, and COG databases, the functional annotations for these proteins were visualized in a Venn diagram (Figure S4). Compared to the control group, the number of differentially expressed proteins (DEPs) during the hypoxia-reoxygenation process was 153, 227, and 172, respectively, with 51, 86, and 60 proteins being upregulated and 102, 141, and 112 proteins being downregulated, respectively. This pattern indicates an initial upregulation followed by a subsequent downregulation (Figure 4A). The Venn diagram further illustrated that 21 proteins were consistently active across the whole hypoxia-reoxygenation procedures, while 91, 143, and 97 proteins were uniquely active in the hypoxia, 6-h reoxygenation, and 12-h reoxygenation phases, respectively (Figure 4B). KEGG enrichment analysis of DEPs revealed distinct pathway enrichments at each stage. During the hypoxic phase, pathways related to fatty acid metabolism and homologous recombination were significantly enriched, which are associated with metabolism and genetic information processing (Figure 4C). At the 6-h reoxygenation stage, apoptosis signaling, glycerolipid metabolism, and RNA transport pathways were notably enriched (Figure 4D). However, at the 12-h reoxygenation stage, no significantly enriched KEGG pathways were observed under a *p*-value threshold of <0.05.

### 2.5. Integrated Negative miRNA-mRNA-Protein Regulatory Network Analysis

Based on microRNA sequencing and mRNA sequencing, we identified differentially expressed miRNAs and mRNAs between the two groups or samples. Subsequently, we explored the regulatory relationships between differentially expressed miRNAs and mRNAs, focusing on the interaction of miRNAs with their target mRNAs. KEGG enrichment analysis of miRNA-mRNA pairs was performed for the hypoxia, 6-h reoxygenation, and 12-h reoxygenation groups, respectively (Figure S5). In the hypoxia group, significantly enriched pathways included the MAPK signaling pathway and the HIF-1 signaling pathway, both of which are widely reported to be closely associated with the response to hypoxic stress [38,39]. In the recovery groups, significant enrichment was observed in pathways such as the cell cycle, ECM-receptor interaction, and calcium signaling pathways, which may be related to the promotion of cell repair, hypoxia adaptation, and ion transport during the recovery process in *E. tetradactylum* brain [30,40,41].

**Figure 5:**
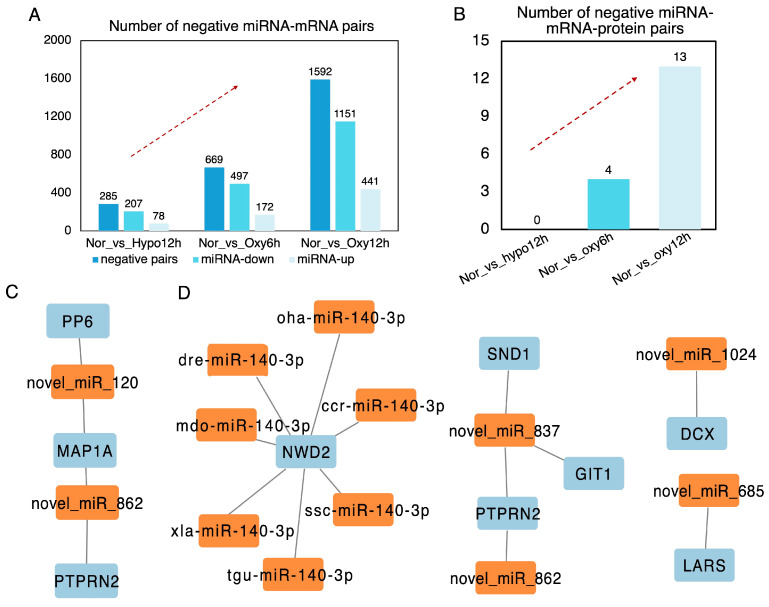
Integrated miRNA-mRNA-Protein analysis in the brain of *Eleutheronema tetradactylum* during hypoxia-recovery processes. (**A**) Number of negative miRNA-mRNA pairs across the hypoxia-recovery phages. (**B**) Number of negative miRNA-mRNA-protein pairs across the hypoxia-recovery processes. (**C**) Interactive network of 2 DEMIs and 3 co-DEGs/DEPs in 4 negative miRNA-mRNA-Protein pairs at 6-h recovery phase. (**D**) Interactive network of 11 DEMIs and 6 co-DEGs/DEPs in 13 negative miRNA-mRNA-Protein pairs at 12-h recovery phase.

As miRNAs generally exert negative regulatory effects on mRNAs, we identified 285, 669, and 1592 miRNA-mRNA pairs across the different hypoxia-recovery processes (Figure 5A, Table S5). We then correlated these findings with proteomic data, identifying 0, 4, and 13 negative miRNA-mRNA-protein pairs in the hypoxia, 6-h reoxygenation, and 12-h reoxygenation groups, respectively (Figure 5B, Table S6). These pairs consisted of 2 DEMIs, 3 co-DEGs/DEPs, and 11 DEMIs (including seven distinct types from different species that actually belong to the same DEMIs), 6 co-DEGs/DEPs in the 6-h and 12-h recovery groups (Figure 5C,D, Table S6). To further explore the associations between these key DEMIs, DEGs, and DEPs, we mapped their functional interaction networks. In the 6-h recovery group, novel_miR_120 and novel_miR_862 were found to interact with microtubule-associated protein 1A (MAP1A), serine/threonine-protein phosphatase 6 (PP6) and receptor-type tyrosine-protein phosphatase N2 (PTPRN2) (Figure 5C). MAP1A is a microtubule-associated protein that stabilizes microtubules, regulates microtubule dynamics, and is involved in processes such as intracellular transport, neurogenesis, and neuronal development, making it essential for maintaining neuronal structure and function [42,43]. PP6 plays a crucial role in regulating various cellular processes, including cell cycle progression, apoptosis, and signal transduction [44,45]. PTPRN2 is implicated in synaptic plasticity, neurotransmitter release, and cell-cell communication, particularly within the central nervous [38]. These functions are potentially closely associated with the initial recovery stages following hypoxia stress in the brain of *E. tetradactylum*.

In the 12-h recovery group, miR_140-3p, which is known to repress targets regulating cell proliferation, apoptosis, senescence, and inflammation, interacted with NACHT and WD repeat domain-containing protein two isoform X2 (NWD2), a protein involved in signal transduction and expressed exclusively in the central nervous system (Figure 5D) [46]. Novel_miR_837 and novel_miR_862 were found to interact with staphylococcal nuclease domain-containing protein 1 (SND1), arf-GAP with GTPase, ANK repeat and PH domain-containing protein 1-like isoform X1 (GIT1), and receptor-type tyrosine-protein phosphatase N2 isoform X1 (PTPRN2) (Figure 5D). SND1 is involved in RNA metabolism, playing a role in RNA processing, mRNA degradation, and the regulation of RNA stability [47]. GIT1 was reported to be involved in synaptic vesicle trafficking, cell signaling, neurodevelopment, and neuronal differentiation, in addition to influencing cell adhesion and morphogenesis [48,49]. The interaction of PTPRN2 in the 12-h recovery group parallels its role observed in the 6-h recovery stage, indicating that this co-expression pair is pivotal throughout the recovery process in the brain of *E. tetradactylum*. Additionally, novel_miR_1024 and novel_miR_685 exhibited unique correlations with neuronal migration protein doublecortin (DCX) and leucine--tRNA ligase, cytoplasmic (LARS), respectively (Figure 5D). DCX is a microtubule-associated protein essential for neuronal migration during brain development, stabilizing microtubules and regulating the cytoskeleton to enable the movement of neurons within the developing brain [50]. LARS is an aminoacyl-tRNA synthetase responsible for attaching the amino acid leucine to its corresponding tRNA molecule, a crucial step in protein synthesis [39,51].

### 2.6. Underlying Metabolism Model During the Hypoxia-Recovery Stage in Eleutheronema tetradactylum Brain

**Figure 6:**
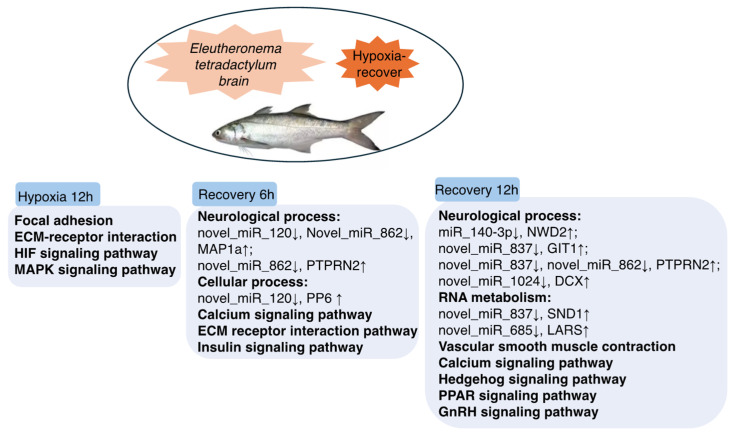
Regulatory model of *Eleutheronema tetradactylum* brain in response to hypoxia-recovery stress.

Through an integrated miRNA-mRNA analysis, we identified key KEGG pathways potentially playing major roles at different stages during hypoxia-recovery processes (Figure S5, Figure 6). At the 12-h hypoxia stage, the pathways Focal Adhesion (ko04510), ECM-Receptor Interaction (ko04512), HIF-1 Signaling Pathway (ko04066), and MAPK Signaling Pathway (ko04010) were found to be closely associated with the physiological response to hypoxia and its regulation [25,31,40,52]. During the 6-h recovery phase, the primary pathways involved appear to be the Calcium Signaling Pathway (ko04020) and the Insulin Signaling Pathway (ko04910). Notably, the ECM-Receptor Interaction (ko04512) pathway, which was significantly enriched during hypoxia, also showed significant enrichment during this 6-h recovery stage. At the 12-h recovery stage, the Vascular Smooth Muscle Contraction (ko04270), Hedgehog Signaling Pathway (ko04340), PPAR Signaling Pathway (ko03320), and GnRH Signaling Pathway (ko04912) are likely to be involved in the repair process. Additionally, the Calcium Signaling Pathway (ko04020) was significantly enriched, suggesting its potential involvement throughout the reoxygenation process.

Furthermore, through miRNA-mRNA-protein correlation analysis, we identified key miRNAs and co-DEGs/DEPs at each of the three stages of hypoxia and reoxygenation. At the hypoxia stage, we did not detect any negative miRNA-mRNA-protein interactions, potentially due to a delay in protein regulation; further investigation was required. At the 6-h and 12-h recovery stages, negative regulatory miRNA-mRNA pairs were characterized by downregulated miRNAs and upregulated mRNAs (Table S6). During the 6-h recovery phase, two novel miRNAs and their corresponding co-DEGs/DEPs were identified. At the 12-h recovery stage, five miRNAs, including one known miRNA and four novel miRNAs, were found to have significant regulatory roles (Figure 6). These novel miRNAs have been relatively underexplored in previous studies, and our findings suggest that they may play crucial roles at different stages of hypoxia recovery in fish brains. However, further research is required to confirm this hypothesis. In conclusion, this study proposes a model predicting the key regulatory pathways across various stages of hypoxia-recovery in *E. tetradactylum* brain. Additionally, it highlights the identification of both novel and known miRNAs, as well as their associated regulatory co-DEGs/DEPs at different hypoxia-recovery stages. These findings may serve as promising biomarkers for advancing research on hypoxia-recovery mechanisms in fish.

## 3. Materials and Methods

### 3.1. Threadfin Fish Culture and Hypoxia-Recovery Experiments

A total of 120 healthy juvenile four-finger threadfin fish (*Eleutheronema tetradactylum*), uniformly sized at 9–10 cm in length, were purchased from a commercial aquaculture farm in Shanwei, Guangdong Province, China. The fish were randomly distributed into six 50 L plastic tanks, with three tanks designated as control groups and three as experimental groups, each containing 20 fish. The fish were acclimated for one week to ensure their physiological stability. In the control groups, dissolved oxygen (DO) levels were maintained at 6.5 mg/L using continuous aeration. In the experimental groups, a hypoxia-recovery treatment was applied. Hypoxia challenge was induced by shutting off the water flow in the tanks and introducing a gas mixture of high-purity nitrogen (99.99%) and oxygen. The dissolved oxygen (DO) levels were closely monitored using a DO meter (YSI professional plus, Yellow Springs, OH, USA) and maintained at 2.0 mg/L by sealing the tanks with plastic wrap. Samples were collected after maintaining hypoxia for 12 h. The recovery process was then initiated by resuming normal aeration and restoring DO levels to 6.5 mg/L. Samples from the experimental groups were collected at 6 h and 12 h after reoxygenation. Concurrently, samples from the control groups were also collected. For sample processing, fresh brain tissues were rinsed 2 times with pre-cooled 1 × phosphate-buffered saline (PBS, Biosharp, Hefei city, China) at 4 °C, frozen in liquid nitrogen, and stored for subsequent multi-omics sequencing. For histopathological analysis, fresh brain samples were fixed in 4% paraformaldehyde (Biosharp, China, BL539A). All experiments were in accordance with the Chinese Guidelines for Ethical Review of Laboratory Animals and Welfare (GB/T 35892–2018) [53].

### 3.2. mRNAome Sequencing and Data Processing

The brain samples of *Eleutheronema tetradactylum* were retrieved from liquid nitrogen, and RNA was extracted using the TRIzol Reagent (Life Technologies, Carlsbad, CA, USA), which followed the standard protocol. RNA concentration and purity were assessed using the NanoDrop 2000 (Thermo Fisher Scientific, Wilmington, DE, USA). RNA integrity was evaluated using the RNA Nano 6000 kit on the Agilent Bioanalyzer 2100 system (Agilent Technologies, Santa Clara, CA, USA). Twelve sequencing libraries (Nor-1, Nor-2, Nor-3, Hypo12h-1, Hypo12h-2, Hypo12h-3, Oxy6h-1, Oxy6h-2, Oxy6h-3, Oxy12h-1, Oxy12h-2, Oxy12h-3) were then constructed according to the instructions provided with the Hieff NGS Ultima Dual-mode mRNA Library Prep Kit for Illumina (Yeasen Biotechnology, Shanghai, China), and the quality of the libraries was evaluated on the Agilent Bioanalyzer 2100 system. The libraries were subsequently sequenced on the Illumina NovaSeq platform to generate 150 bp paired-end reads in accordance with the manufacturer’s guidelines.

The generated raw data were initially processed using FastQC (version 0.12.1) and Trimmomatic (version 0.39) to obtain clean data [54,55]. The clean data were then aligned to the reference genome using Hisat2 (version 2.2.1) to obtain the alignment information [56]. Subsequently, StringTie (version 2.2.1) was used to assemble the mapped reads and reconstruct the transcriptome for downstream analysis [57]. Expression levels of transcripts and genes were quantified using Fragments Per Kilobase of transcript per Million fragments mapped (FPKM) via the maximum flow algorithm in StringTie. Principal Component Analysis (PCA) was performed to evaluate the variance across samples [58]. Differential expression analysis was conducted using DESeq2 (version 1.30.1), where genes with an FDR < 0.01 and fold change (FC) ≥ 1.5 were considered differentially expressed [59]. KEGG pathway enrichment of differentially expressed genes (DEGs) was performed using the clusterProfiler software (version 4.4.4), with a significance cutoff of *p*-value < 0.05 [60]. To categorize the gene expression trends throughout the hypoxia-recovery process, a time-series analysis was conducted using the Short Time-series Expression Miner (STEM) software (version 1.3.13) [61]. A *p*-value < 0.05 was considered statistically significant, and the number of expression trends was set to 20.

### 3.3. miRNAome Sequencing and Data Analysis

Twelve microRNA (miRNAs) libraries (Nor-1, Nor-2, Nor-3, Hypo12h-1, Hypo12h-2, Hypo12h-3, Oxy6h-1, Oxy6h-2, Oxy6h-3, Oxy12h-1, Oxy12h-2, Oxy12h-3) were prepared following the standard protocol of the VAHTS Small RNA Library Prep Kit for Illumina v2 (NR811-02). The libraries were then sequenced on the Illumina NovaSeq X Plus platform. Raw reads with adapters, poly-N sequences, as well as low-quality reads were removed using in-house Perl scripts, with reads shorter than 18 nucleotides or longer than 30 nucleotides were trimmed to generate high-quality clean data for downstream analyses.

Clean reads were aligned with the Silva, GtRNAdb, Rfam, and Repbase databases using the Bowtie package (version 1.1.1) to filter out ribosomal RNA (rRNA), transfer RNA (tRNA), small nuclear RNA (snRNA), small nucleolar RNA (snoRNA), and repetitive sequences [62]. The remaining unannotated reads, potentially contained miRNAs, were subsequently mapped to the reference genome of *E. tetradactylum* using Bowtie. These mapped reads were further compared to the mature miRNA sequences and their flanking regions (upstream 2 nt and downstream 5 nt) in the miRBase database (version 22), allowing for a maximum of one mismatch [63]. Reads matching this criterion were identified as known miRNAs, while unclassified reads were analyzed using miRDeep2 software (version 2.0.5) to predict novel miRNAs [64]. Differentially expressed miRNAs were identified using DESeq2 (version 1.30.1) with thresholds of fold change (FC) ≥ 1.5 and a *p*-value < 0.05 [59]. Target gene prediction was performed using miRanda (version 3.3a) and TargetScan (version 5.0). Predicted target genes were annotated by aligning their sequences to the NR, Swiss-Prot, GO, COG, KEGG, KOG, and Pfam databases with BLAST software (version 2.2.26) [65]. KEGG pathway enrichment analysis for the target genes of differentially expressed miRNAs was conducted using the clusterProfiler package (version 2.2.4), with a significance threshold of *p*-value < 0.05 [60].

### 3.4. Proteome Sequencing and Data Exploration

Proteins were extracted by adding 300 µL of 8 M urea solution to each sample (Nor-1, Nor-2, Nor-3, Hypo12h-1, Hypo12h-2, Hypo12h-3, Oxy6h-1, Oxy6h-2, Oxy6h-3, Oxy12h-1, Oxy12h-2, Oxy12h-3), along with protease inhibitors at 10% of the lysate volume. After centrifugation at 14,000× *g* for 20 min, the supernatant was collected, and protein concentrations were tested using the Bradford method. For each sample, 50 µg of protein was reduced with 200 mM dithiothreitol (DTT) at 37 °C for 1 h, followed by an eightfold dilution with 50 mM ammonium bicarbonate (ABC) buffer. Proteins were digested overnight at 37 °C using trypsin at a trypsin-to-protein ratio of 1:25. The digested peptides were separated using reversed-phase (RP) chromatography according to standard protocols and analyzed by liquid chromatography-tandem mass spectrometry (LC-MS/MS) on a Q Exactive HF-X system (Thermo Fisher Scientific, USA). Lyophilized peptides were reconstituted in 0.1% formic acid and separated on NuPAGE 4–12% Bis-Tris protein gels (Thermo Fisher Scientific, USA) prior to LC-MS/MS. The 40 most abundant precursor ions from full-scan mode were selected for high-energy collision dissociation (HCD) fragmentation.

Raw MS/MS data were analyzed using MaxQuant software (version 1.5.2.8), and the peptide and protein identification false discovery rate (FDR) was set to 1% [66]. Protein quantification was performed using the intensity-based absolute quantification (iBAQ) algorithm, and common contaminants and reverse hits were excluded. A two-tailed t-test was applied to relative protein quantification values between comparison groups, using thresholds of FC ≥ 1.2 and *p*-value < 0.05 to identify differentially expressed proteins (DEPs). Functional annotation of the identified proteins was conducted using GO, KEGG, and COG databases, employing tools such as Python (version 3.6.5), Pandas (version 0.24.2), ggplot2 (version 3.3.3), and DIAMOND (version 2.0.4) [67,68,69]. Venn diagrams illustrating annotation overlap across databases were generated using the Venn diagram package (version 1.6.20) [70]. KEGG pathway enrichment analysis of these proteins was performed using the clusterProfiler package (version 2.0.1) and visualized with ggplot2 (version 3.3.3) [60,68].

### 3.5. Integrative Multi-Omics Analysis of mRNA, miRNA, and Proteins

To investigate the regulatory relationships between differentially expressed mRNAs (DEGs), differentially expressed miRNAs (DEMIs), and differentially expressed proteins (DEPs), an integrated analysis was performed using miRanda (version 3.3a) and targetscan (version 5.0). Given the general negative regulatory effect of miRNAs on mRNAs, we prioritized the investigation of miRNA-mRNA pairs exhibiting inverse expression patterns (i.e., upregulated miRNA-downregulated mRNA and downregulated miRNA-upregulated mRNA). KEGG pathway enrichment analysis of regulated DEGs was conducted using the clusterProfiler package (version 2.2.4), with a significance threshold set at *p*-value < 0.05 [60]. To investigate miRNA-mRNA-protein interactions, the DEPs were matched to the identified miRNA-mRNA pairs. We used KEGG annotation to associate the transcriptome and proteome, where genes and proteins annotated with the same KEGG functional ortholog number were considered as correlated gene-protein pairs, allowing for the identification of miRNA-mRNA-protein interaction triplets. These triplets provided a comprehensive representation of the regulatory relationships across different molecular levels. Finally, Cytoscape software (version 3.10.3) was used to visualize the relationships between DEMIs and DEGs/DEPs, generating an interaction network that facilitates an in-depth understanding of the regulatory mechanisms during the entire hypoxia-recovery process in the brain of *E*. *tetradactylum* [71].

### 3.6. Imaging of Threadfin Fish Brain During the Hypoxia-Recovery Processes

The brain samples of *E. tetradactylum* were retrieved from a 4% paraformaldehyde fixation solution and placed into embedding cassettes with appropriate labels. The cassettes were then processed in a tissue dehydration machine, undergoing dehydration in a series of graded ethanol solutions, followed by clearing in xylene and paraffin infiltration according to the standard protocols. The paraffin-infiltrated tissues were embedded using an embedding machine and cooled on a −20 °C freezing plate. Once the paraffin solidified, the tissue blocks were removed from the cassettes and trimmed. The paraffin blocks were sectioned into 4-μm-thick transverse slices using a microtome. The sections were transferred to a 40 °C water bath, subsequently mounted onto glass slides, and left to air-dry overnight. The slides were subsequently dewaxed with xylene (HEMO-DE) and stained with hematoxylin and eosin (H&E) protocols (Servicebio, Wuhan, China). Coverslips were applied to the slides, which were air-dried at room temperature for 48 h. Fluorescence microscopy was performed to observe and image the samples using a Nikon Eclipse E100 microscope (Nikon, Tokyo, Japan).

## 4. Conclusions

This study presents a comprehensive multi-omics analysis of the brain’s response to hypoxia and subsequent recovery in *Eleutheronema tetradactylum*. The results revealed a complex regulatory network involving differential expression of genes, miRNAs, and proteins. Despite recovery efforts, the brain tissue did not fully return to baseline levels within the 12-h recovery period, suggesting the need for longer recovery phases for complete restoration. Multi-omics interaction analysis revealed key regulatory pathways and essential miRNA-mRNA-protein interactions across the hypoxia-recovery stages. During the 6-h recovery phase, novel miRNAs such as novel_miR_120 and novel_miR_862, and their regulation of co-DEGs/DEPs like MAP1A, PTPRN2, and PP6 were identified. In the 12-h recovery phase, miR_140-3p, novel_miR_837, novel_miR_1024, novel_miR_862, and novel_miR_685, along with their regulation of co-DEGs/DEPs such as NWD2, GIT1, PTPRN1, DCX, SND1, and LARS, were found to play significant roles. Our findings provide valuable insights into the molecular mechanisms of hypoxia recovery and establish a foundation for future investigations underlying hypoxia resilience and recovery in fish.

## Data Availability

Data will be made available on request.

## Supplementary Materials

The following supporting information can be downloaded at https://www.mdpi.com/article/10.3390/ijms26041703/s1.

## Abbreviations

ROS	Reactive oxygen species
DO	Dissolved oxygen
PBS	Phosphate-buffered saline
FPKM	Fragments Per Kilobase of transcript per Million fragments mapped
PCA	Principal Component Analysis
STEM	Short Time-series Expression Miner
rRNA	Ribosomal RNA
tRNA	Transfer RNA
snRNA	Small nuclear RNA
snoRNA	Small nucleolar RNA
miRNA	Micro RNA
FDR	False discovery rate
DEGs	Differentially expressed genes
DEMIs	Differentially expressed microRNAs
DEPs	Differentially expressed proteins
MAPK	Mitogen-activated protein kinase
GUCA1a	Guanylate cyclase activator 1a
LOC111585952	Potassium voltage-gated channel subfamily V member 2-like
LOC110002783	Retinal cone rhodopsin-sensitive cGMP 3′,5′-cyclic phosphodiesterase. subunit gamma
LOC115047007	Hemoglobin embryonic subunit alpha-like
Hbbe1.1	Hemoglobin subunit beta-2-like
MAP1a	Microtubule-associated protein 1A
PP6	Serine/threonine-protein phosphatase 6
PTPRN2	Receptor-type tyrosine-protein phosphatase N2
NWD2	NACHT and WD repeat domain-containing protein 2 isoform X2
SND1	Staphylococcal nuclease domain-containing protein 1
GIT1	Arf-GAP with GTPase, ANK repeat, and PH domain-containing protein 1-like isoform X1
DCX	Neuronal migration protein doublecortin
LARS	Leucine--tRNA ligase, cytoplasmic
