# Supplementary material for: Molecular Modulation of Threadfin Fish Brain to Hypoxia Challenge and Recovery Revealed by Multi-Omics Profiling
Source: Int J Mol Sci. 2025 Feb 17;26(4):1703. doi: 10.3390/ijms26041703 (PMC11855007; doi:10.3390/ijms26041703)
Supplement: Supplementary file 1 [file ijms-26-01703-s001.zip › 1-Supplementary figures.pdf]

Molecular Modulation of Threadfin Fish Brain to Hypoxia Challenge and Recovery Revealed by Multi-Omics Profiling

Xiaoli Ma [1,2] and Wen-Xiong Wang [1,2,*]

[1] School of Energy and Environment and State Key Laboratory of Marine Pollution, City University of Hong Kong, Kowloon, Hong Kong, China; xiaolima@cityu.edu.hk
[2] Research Centre for the Oceans and Human Health, City University of Hong Kong Shenzhen Research Institute, Shenzhen 518057, China
* Correspondence: wx.wang@cityu.edu.hk

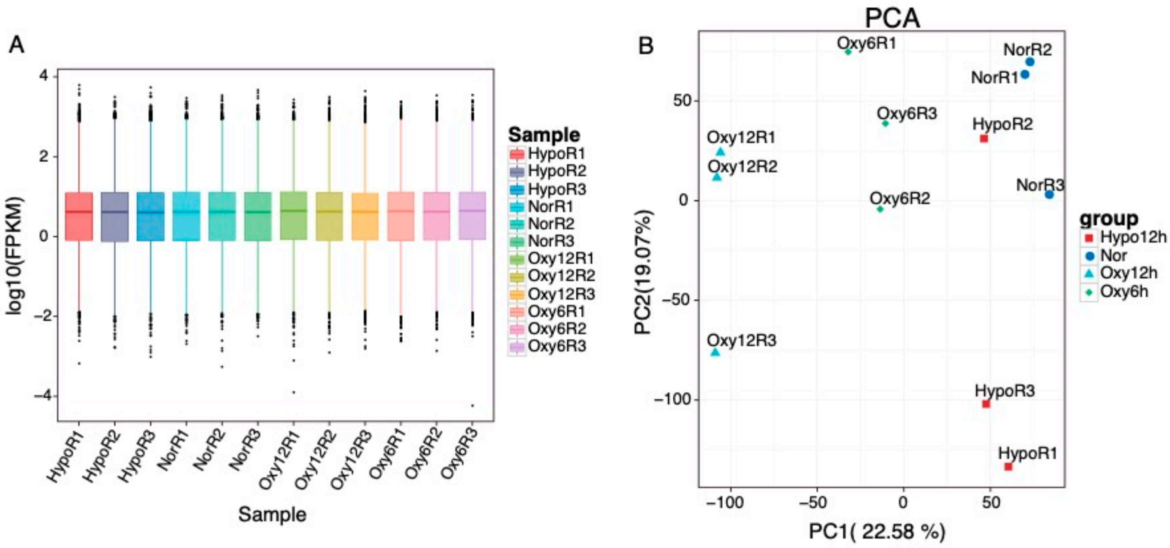

Figure S1. Overview of mRNA expression in the brain of *Eleutheronema tetradactylum*. (A) Box plots illustrating gene expression levels across various samples. The x-axis represents individual samples, while the y-axis shows the logarithmic scale of gene expression values (FPKM). The overall expression variability across samples is depicted. (B) Principal component analysis (PCA) plot showing the relationships among samples. The axes represent different principal components, with the percentages indicating their contributions to the variation among samples. Each point represents a sample, and different groups are distinguished by colors and shapes.

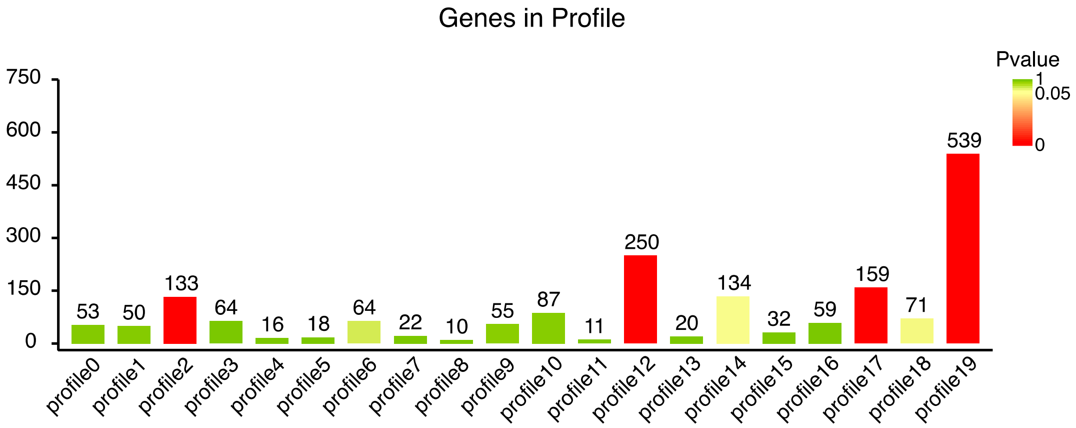

Figure S2. Number of differentially expressed genes (DEGs) across 20 expression profiles in *Eleutheronema tetradactylum.*

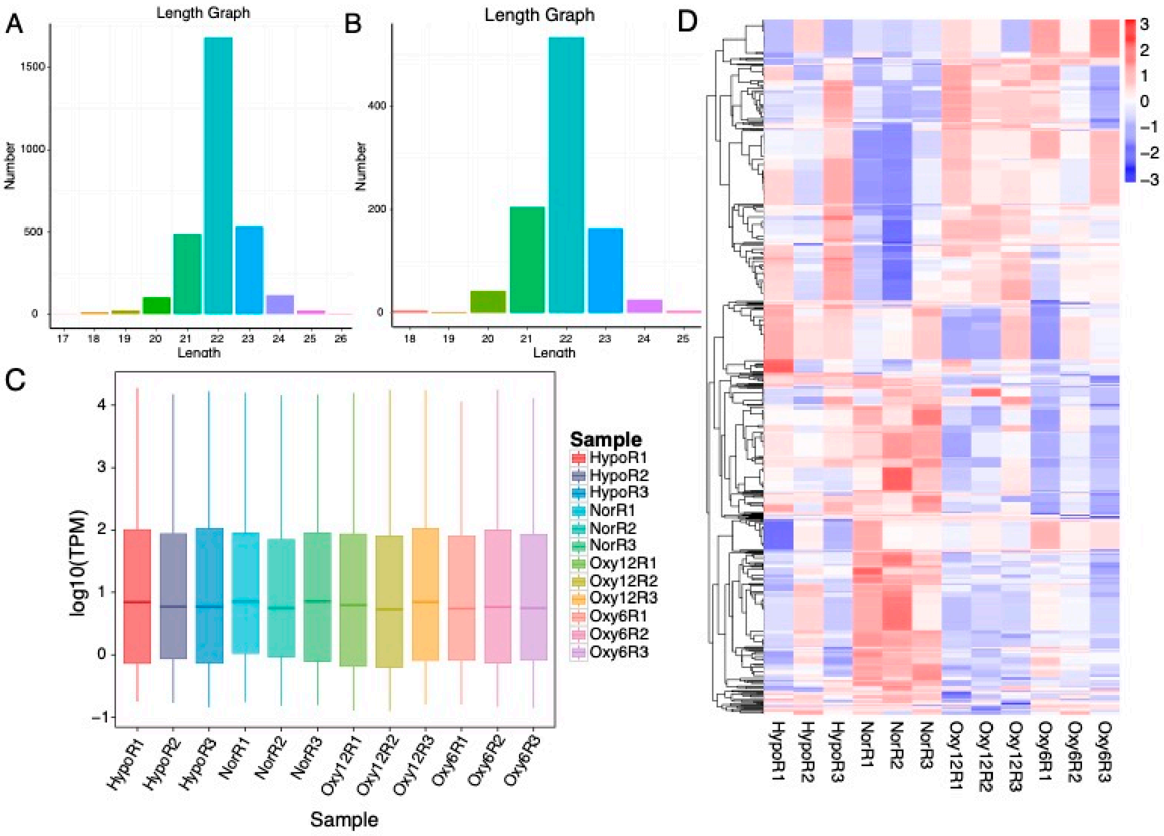

Figure S3. Overview of miRNA expression in the brain of *Eleutheronema tetradactylum*. (A) Length distribution of known miRNAs. The x-axis indicates miRNA lengths, and the y-axis represents the count of miRNAs for each specific length. (B) Length profiles of newly predicted miRNAs. The x-axis represents miRNA lengths, and the y-axis shows the number of miRNAs of each length. (C) Box plots showing the overall distribution of miRNA expression across samples. (D) Cluster diagram comparing different samples. The x-axis represents sample names.

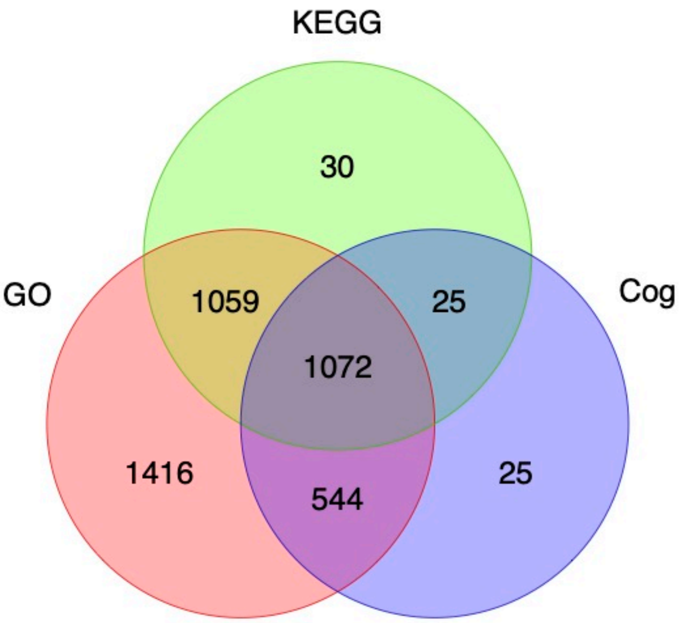

Figure S4. Venn diagram of functional annotations for identified proteins based on GO, KEGG, and COG databases.

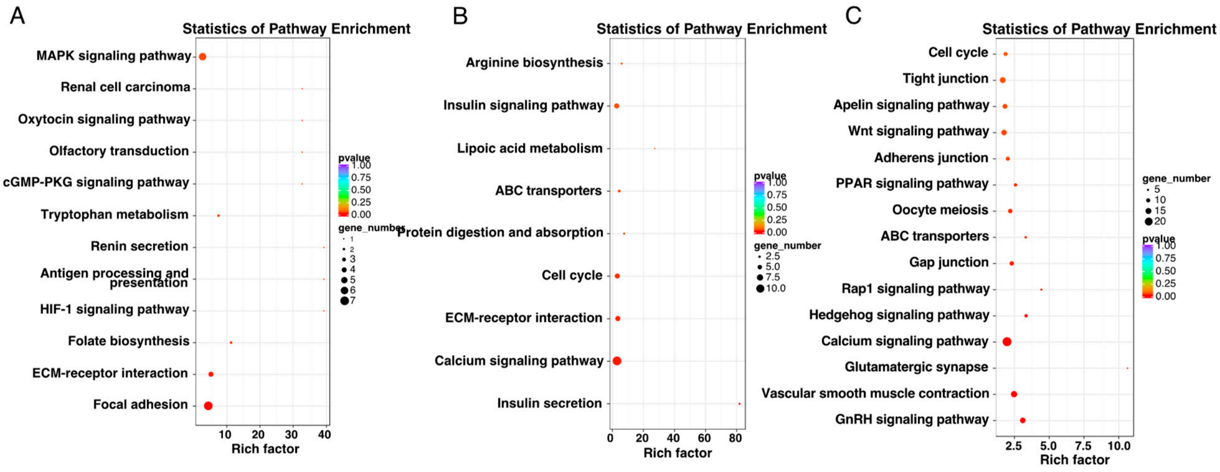

Figure S5. KEGG pathway enrichment of the miRNA-mRNA pairs after (C) 6-hour hypoxia, (D) 6-hour recovery, and (E) 12-hour recovery.
